# Incidence and risk factors of venous thrombotic events in patients with interstitial lung disease during hospitalization

**DOI:** 10.1186/s12959-023-00458-7

**Published:** 2023-02-10

**Authors:** Haishuang Sun, Min Liu, Xiaoyan Yang, Linfeng Xi, Wenqing Xu, Mei Deng, Yanhong Ren, Wanmu Xie, Huaping Dai, Chen Wang

**Affiliations:** 1 Department of Respiratory Medicine, The First Hospital of Jilin University, Changchun, 130021 China; 2 National Center for Respiratory Medicine, National Clinical Research Center for Respiratory Diseases, Institute of Respiratory Medicine, Chinese Academy of Medical Sciences, Department of Pulmonary and Critical Care Medicine, Center of Respiratory Medicine, China-Japan Friendship Hospital, 100029 Beijing, China; 3 Chinese Academy of Medical Sciences and Peking Union Medical College, Beijing, 100730 China; 4 Department of Radiology, China-Japan Friendship Hospital, Beijing, 100029 China

**Keywords:** Idiopathic pulmonary fibrosis, Venous thromboembolism, Pulmonary embolism, Deep vein thrombosis

## Abstract

**Background:**

Studies on the incidence of venous thromboembolism (VTE) events in patients with interstitial lung disease (ILD) are limited and the results are inconsistent. The aim of this research was to investigate the incidence and risk factors of VTE in ILD during hospitalization.

**Materials and methods:**

In this retrospective, cross-sectional, observational study, a total of 5009 patients diagnosed with ILD from January 2016 to March 2022 in our hospital were retrospectively included. In ILD patients, VTE including pulmonary thromboembolism (PTE) and deep vein thrombosis (DVT) were screened from the electronic medical record system. Diagnosis of PTE and DVT were performed by CT pulmonary angiography (CTPA), CTV or ultrasound. And then the incidence and risk factors of VTE in different types of ILD were assessed.

**Results:**

Among 5009 patients with ILD, VTE was detected in 129 (2.6%) patients, including 15(0.3%) patients with both PTE and DVT, 34 (0.7%) patients with PTE and 80 (1.6%) patients with DVT. 85.1% of patients with APE were in the intermediate-low risk group. The incidence of VTE in Anti-Neutrophil Cytoplasmic Antibodies -associated vasculitis related ILD (ANCA-AV-ILD), hypersensitivity pneumonitis and idiopathic pulmonary fibrosis (IPF) respectively was 7.9% and 3.6% and 3.5%. In patients with connective tissue disease-associated ILD (CTD-ILD), the incidence of VTE, DVT, PTE, combined PTE and DVT respectively was 3.0%, 2.3%, 0.4% and 0.3%. Among the various risk factors, different ILD categories, age ≥ 80 years (OR 4.178, 95% CI 2.097–8.321, *P* < 0.001), respiratory failure (OR 2.382, 95% CI 1.533–3.702, *P* < 0.001) and varicose veins (OR 3.718, 95% CI 1.066–12.964, *P* = 0.039) were independent risk factors of VTE. The incidence of VTE in patients with ILD increased with the length of time in hospital from 2.2% (< 7 days) to 6.4% (> 21 days).

**Conclusion:**

The incidence of VTE during hospitalization in ILD patients was 2.6%, with a 1.6% incidence of DVT, higher than the 0.7% incidence of PTE. Advanced age, ILD categories, respiratory failure and varicose veins as independent risk factors for the development of VTE should be closely monitored.

## Introduction

Venous thromboembolism (VTE) including pulmonary thromboembolism (PTE) and deep vein thrombosis (DVT) has an annual incidence of 1:1000 [1] and PTE is one of the biggest threats to healthcare worldwide. As a complex multifactorial disease, VTE is associated with genetic predisposition to thrombosis and environmental exposures [2, 3]. Interstitial lung disease (ILDs) refers to a heterogeneous group of diseases characterized by varying degrees of interstitial inflammation and fibrosis [4, 5]. According to the 2013 classification update by the American Thoracic Society (ATS)/European Respiratory Society (ERS), ILDs are distinguished as idiopathic interstitial pneumonias (IIPs), rare IIPs and unclassifiable IIPs. Several preclinical and clinical studies demonstrated that a coagulation cascade can be observed in animal models of pulmonary fibrosis and in patients with idiopathic pulmonary fibrosis (IPF) [6–13]. The exact cause of the correlation between IPF and VTE is unknown, however, an association between IPF and increased risk of VTE has been reported. The risk of VTE in IPF decedents was 34% higher than in the background population, and 44% and 54% greater than among decedents with chronic obstructive pulmonary disease and lung cancer, respectively [14]. Moreover, those with IPF and VTE died at a younger age than those with IPF alone. A meta-analysis by Boonpheng et al. [15] showed that the pooled risk ratio of VTE in IPF was 2.1 (95% CI1.3–3.5), while Margaritopoulos et al. [16] reviewed a VTE incidence of 2% in IPF patients, two-fold higher than healthy individuals. Moreover, ILD is one of the most common lung manifestations in connective tissue disease (CTD) [17]. The risk of DVT was 2–3 times higher in patients with CTD-ILD than within the non-CTD-ILD population. However, incidence and risk factors of VTE in Chinese patients with different types of ILD remains unclear, we aimed to investigate the epidemiological relationship between various ILDs and VTE.

## Materials and methods

### Study cohort and design

**Fig. 1:**
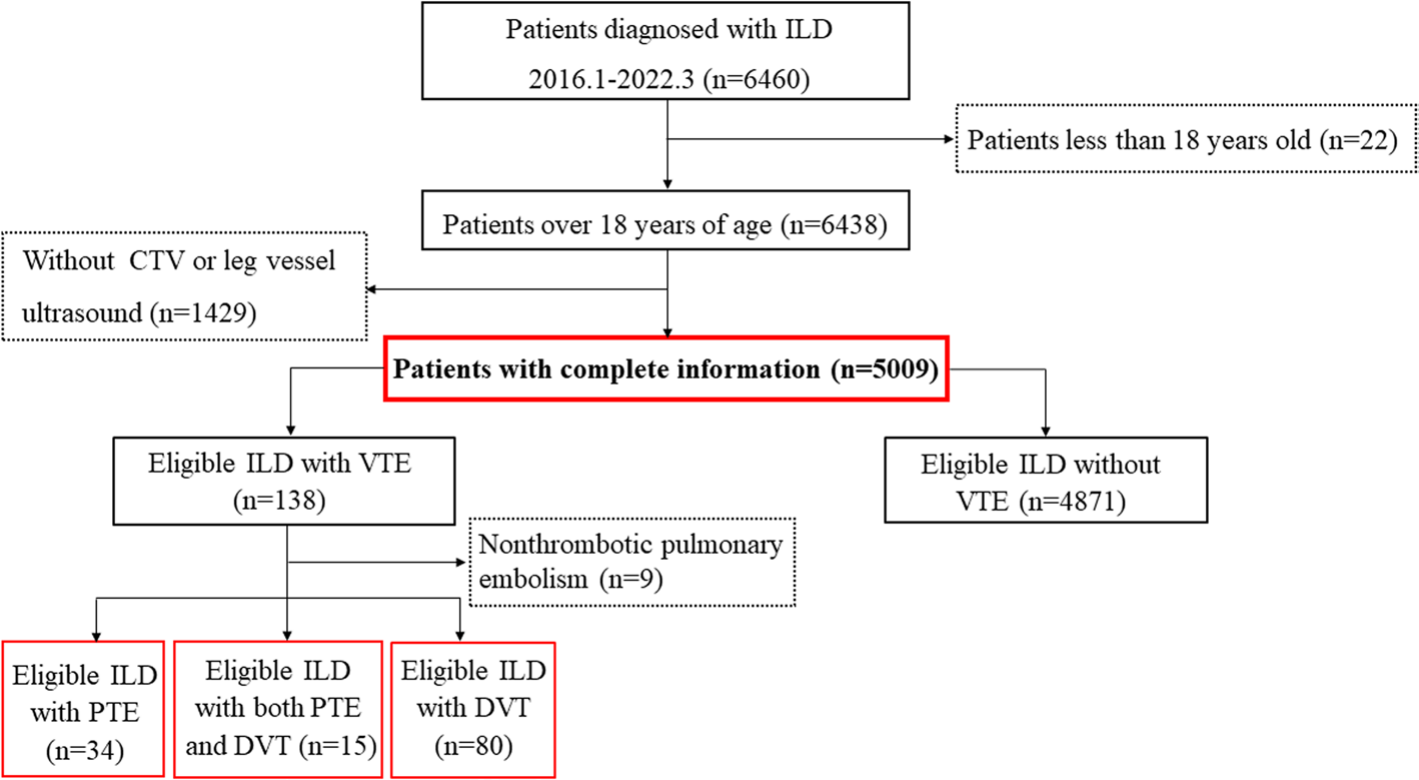
Screening flow chart. ILD, interstitial lung disease; CTPA, computed tomography pulmonary angiogram; CTV, CT venography; VTE, venous thromboembolism; PTE, pulmonary thromboembolism; DVT, deep vein thrombosis

This was a retrospective cross-sectional study in our hospital. The study was performed with approval from the Chinese Clinical Trials Registry Center (http://www.chictr.org/en/; Registration number ChiCTR-OCH-14004929) and was approved by Ethics Board of China-Japan Friendship Hospital Committee (No.2019–123-K85). Figure 1 was the flowchart detailing how participants were selected and how the research was conducted. Firstly, we reviewed the electronic medical record system of our hospital from January 2016 to March 2022 and screened 5009 cases with ILD from the discharge medical records. Second, based on the records in the discharge medical charts,129 cases were diagnosed with PTE or DVT during hospitalization. According to the risk stratification recorded by the guidelines of the European Society of Cardiology (ESC) and the European Respiratory Society (ERS) in 2019 [18], taking into account a combination of clinical, imaging, and laboratory indicators, patients with PTE were classified into low-risk, intermediate-high risk, intermediate-low risk and high risk groups based on predisposing factors for VTE including strong risk factors (myocardial infarction, atrial fibrillation, trauma/surgery), moderate risk factors (autoimmune diseases, respiratory failure, lung infection, urinary tract infections, inflammatory bowel disease, malignancies) and weak risk factors (diabetes, hypertension, varicose veins) [18]. Patients with pulmonary arterial sarcoma, Takayasu arteritis and nonthrombotic pulmonary embolism in the discharge medical charts were excluded from our study. The primary endpoint is the incidence of both symptomatic and asymptomatic VTE in ILD patients during hospitalization.

### HRCT, CT pulmonary angiography (CTPA) and CT venography (CTV)

All patients underwent HRCT on the multi-layer spiral CT device (Optima CT660, GE Healthcare; Lightspeed VCT/64, GE Healthcare; Toshiba Aquilion ONE TSX-301C/320; Philips iCT/256; Siemens FLASH Dual Source CT). HRCT scanning protocol was spiral mode with the acquisition and reconstruction parameters as follows: tube voltage of 100–120 kV, tube current of 100–300 mAs, table speed of 39.37 mm/s, gantry rotation time of 0.8 s, section thickness of 0.625–1 mm, and reconstruction increment of 1–1.25 mm. All images were acquired with the patient at the end of inspiration and in supine position, and scans ranged from the lung apex to the lung level.

CTPA was performed in the craniocaudal direction with multidetector CT scanners (Lightspeed VCT/64, GE Healthcare; Toshiba Aquilion ONE TSX-301C/320; Philips iCT/256; SOMATOM Definition Dual Source CT). The whole chest was craniocaudally scanned from lung apex to the lowest hemidiaphragm during a single breath-hold. Scan parameters were as follows: tube voltage of 100–120 kV, tube current of 100–300 mAs, section thickness of 0.625–1 mm, table speed of 39.37 mm/s, gantry rotation time of 0.8 s, and reconstruction increment of 1–1.25 mm. A soft tissue reconstruction kernel was used. A mechanical injector was used for intravenous bolus injection of iopromide (Ultravist, 370 mg/ml, Bayer Schering Pharma) at a flow rate of 5.0 ml/s. For optimal intraluminal contrast enhancement, the automatic bolus-tracking technique had the region of interest positioned at the level of the main pulmonary artery with a threshold of 100 HU predefined threshold, and a fixed delay of 5 s was employed for data acquisition. CTV was performed in 120 s after CTPA from iliac vein to lower leg vein, section thickness of 1 mm and reconstruction increment of 2–2.5 mm.

### ILD Evaluation

According to the American Thoracic Society/European Respiratory Society guidelines [5], ILD was classified into the following categories: IPF, nonspecific interstitial pneumonia (NSIP), cryptogenic organized pneumonia (COP), hypersensitivity pneumonitis (HP), Anti-Neutrophil Cytoplasmic Antibodies -associated vasculitis related ILD (ANCA-AV-ILD), CTD-ILD, and in our study, the rest cases were classified as other categories of ILD. Meanwhile, CTD-ILD was classified as idiopathic inflammatory myopathies related ILD (IIM-ILD), Sjogren’s syndrome related ILD (SS-ILD), rheumatoid arthritis related ILD (RA-ILD), and systemic lupus erythematosus related ILD (SLE-ILD), and others were classified as other CTD-ILD (OCTD-ILD) according to the etiology.

### VTE evaluation

PTE was diagnosed by two radiologists on CTPA, which was characterized by a hypointense filling defect within the pulmonary artery, partially or completely surrounded by opaque blood flow or a complete filling defect [19]. The diagnostic criteria for DVT are the presence of intraluminal thrombus, incomplete compressibility in 2D mode or vascular filling defect with residual blood flow in CTV or color Doppler mode under ultrasound guidance.

### Statistical analysis

All statistical analyses were performed with SPSS software (version 24.0, IBM Corporation, Armonk, NY, USA) and R (version 3.6.0, R Foundation for Statistical Computing, Vienna, Austria). We counted patients diagnosed with ILD from 2016 to 2022 and calculated the incidence of concomitant PTE and DVT, PTE, DVT and VTE (per 100). Continuous variables conforming to a normal distribution are expressed as mean ± standard deviation (SD) and non-normally distributed continuous variables are expressed as median and interquartile range (IQR). Binary logistics regression model was used for the prediction of independent risk factors of VTE. *P*-values were bilateral, the result with *P* < 0.05 was defined as a statistically significant.

## Results

### Demographics of ILD

A total of 5009 patients with ILD were included in this study. The median age of the total population was 62 years (interquartile range (IQR), 53 to 70 years), with a predominance of patients over 60 years (57.3%). According the diagnosis on the electronic medical record system, ILD included IPF in 510 patients (10.2%), NSIP in 407 patients (8.1%), CTD-ILD in 1576 patients (31.5%), COP in 534 patients (10.7%), HP in 250 patients (5.0%), ANCA-AV-ILD in 63 patients (1.3%) and other ILD in 1669 patients (33.3%), respectively. In this elder population, the highest incidence was 31.4% at the age of 60–69 years. It was slightly more common in male than in female (51.6% vs 48.4%). Autoimmune diseases, hypertension and pulmonary infections were the three most common comorbidities with the incidence of 30.6%, 24.7% and 24.1%, respectively. Myocardial infarction and respiratory failure occurred most frequently in patients with IPF with incidence rates of 2.7% and 17.3%. Detailed demographic information was shown in Table 1.

**Table 1:** Demographic characteristics of different types of ILD

Characteristics	Total, *n* = 5009(%)	IPF, *n* = 510 (%)	NSIP, *n* = 407, (%)	CTD-ILD, *n* = 1576 (%)	COP, *n* = 534 (%)	HP, *n* = 250 (%)	ANCA-AV-ILD, *n* = 63 (%)	Others, *n* = 1669 (%)
**Age, Median (IQR) years**	62(53–70)	67(61–72)	61(53–69)	59(50–67)	60(51–68)	58(47–66)	68(59–75)	64(54–72)
< 60	2138(42.7)	103(20.2)	181(44.5)	801(50.8)	262(49.1)	131(52.4)	16(25.4)	644(38.6)
60–69	1573(31.4)	209(41.0)	132(32.4)	486(30.8)	162(30.3)	81(32.4)	20(31.7)	483(28.9)
70–79	984(19.6)	159(31.2)	80(19.7)	240(15.2)	88(16.5)	36(14.4)	19(30.2)	362(21.7)
≥ 80	314(6.3)	39(7.6)	14(3.4)	49(3.1)	22(4.1)	2(0.8)	8(12.7)	180(10.8)
**Male**	2585(51.6)	426(83.5)	204(50.1)	493(31.3)	274(51.3)	112(44.8)	35(55.6)	1041(62.4)
**Predisposing factors**								
**Strong risk factors**								
Myocardial infarction	82(1.6)	14(2.7)	7(1.7)	11(0.7)	5(0.9)	6(2.4)	1(1.6)	38(2.3)
Atrial fibrillation	98(2.0)	7(1.4)	13(3.2)	12(0.8)	13(2.4)	2(0.8)	1(1.6)	50(3.0)
Trauma/surgery	47(0.9)	2(0.4)	2(0.5)	22(1.4)	5(0.9)	0(0)	1(1.6)	15(0.9)
**Moderate risk factors**								
Autoimmune diseases	1532(30.6)	3(0.6)	4(1.0)	1576(100.0)	4(0.7)	1(0.4)	0(0)	13(0.8)
Respiratory failure	623(12.4)	88(17.3)	29(7.1)	152(9.6)	46(8.6)	22(8.8)	3(4.8)	283(17.0)
Lung infection	1205(24.1)	126(24.7)	75(18.4)	393(24.9)	145(27.2)	38(15.2)	20(31.7)	408(24.4)
Urinary tract infections	51(1.0)	4(0.8)	4(1.0)	22(1.4)	7(1.3)	2(0.8)	0(0)	12(0.7)
Inflammatory bowel disease	22(0.3)	0(0)	1(0.2)	6(0.4)	3(0.6)	1(0.4)	0(0)	11(0.7)
Malignancies	253(5.1)	26(5.1)	18(4.4)	40(2.5)	39(7.3)	6(2.4)	2(3.2)	122(7.3)
**Week risk factors**								
Diabetes	806(16.1)	118(23.1)	62(15.2)	214(13.6)	98(18.4)	31(12.4)	10(15.9)	273(16.4)
Hypertension	1236(24.7)	122(23.9)	101(24.8)	315(20.0)	140(26.2)	66(26.4)	11(17.5)	481(28.8)
Varicose veins	35(0.7)	2(0.4)	0(0)	11(0.7)	5(0.9)	1(0.4)	0(0)	16(1.0)

*ILD* interstitial lung disease, *IPF* idiopathic pulmonary fibrosis, *NSIP* nonspecific interstitial pneumonia, *CTD-ILD* connective tissue disease-associated ILD, *COP* cryptogenic organized pneumonia, *HP* hypersensitivity pneumonitis, *ANCA-AV-ILD* Anti-Neutrophil Cytoplasmic Antibodies -associated vasculitis related ILD, *IQR* interquartile range

### Incidence of VTE in patients with ILD

**Fig. 2:**
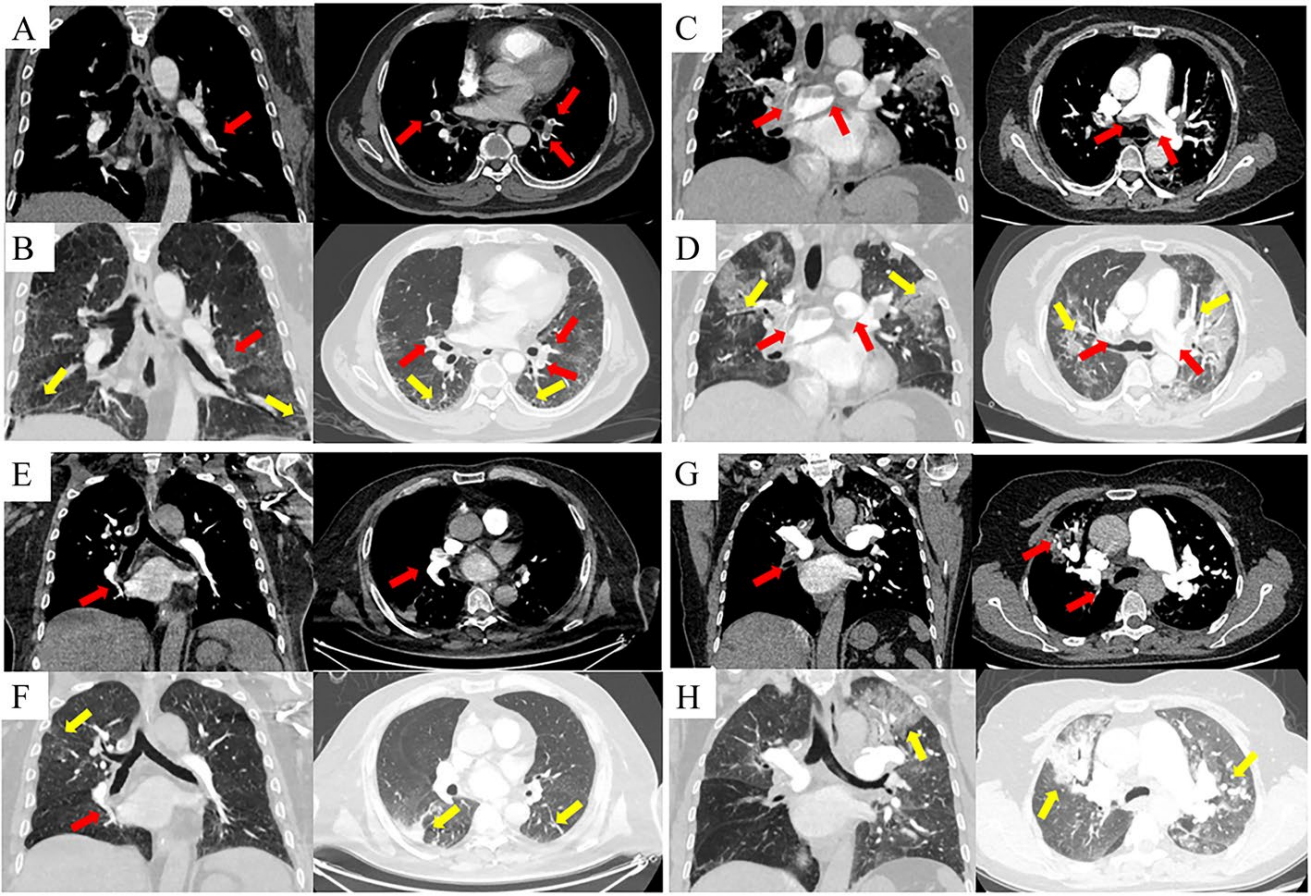
Examples of imaging presentations of four patients with pulmonary thromboembolism (PTE). **A** A 66-year-old male with a diagnosis of idiopathic pulmonary fibrosis. CTPA showed pulmonary artery embolism in the middle lobe of the right lung and lower lobe of both lungs, and (**B**) HRCT exhibited subpleural honeycombing and reticular pattern with ground glass opacity changes. **C** A 57-year-old female with a diagnosis of cryptogenic organized pneumonia. CTPA showed embolism of the right and left main pulmonary arteries, and (**D**) HRCT exhibited ground glass opacity changes and mosaic attenuation. **E** A 53-year-old male with a diagnosis of HP and was in the intermediate-low risk group for pulmonary embolism. A filling defect was seen in the dorsal segment of the right lower lobe, and (**F**) HRCT showed thickened lobular septa, multiple small nodules in both lungs and irregular solid changes in the right upper lung. **G** A 59-year-old female with a diagnosis of Anti-Neutrophil Cytoplasmic Antibodies -associated vasculitis related ILD combined with pulmonary embolism in the intermediate-low risk group. CTPA demonstrated occlusion of the right lower pulmonary artery trunk and widening of the pulmonary arteries, and (**H**) HRCT showed multiple ground glass opacity in both lungs. Red arrows represent pulmonary emboli and yellow arrows represent ILD changes (honeycombing, reticular, ground glass opacity, etc.). CTPA, CT pulmonary arteriography; HRCT, high resolution computed tomography; ILD, interstitial lung disease

129(2.6%) patients developed VTE out of 5009 ILD patients during hospitalization. The incidence of DVT, PTE, concomitant PTE and DVT respectively was 1.6%, 0.7% and 0.3%. There were 47 patients with acute PTE (APE) and 2 patients with chronic PTE (CPE) in patients with PTE. Figure 2 demonstrated patients with IPF, COP, HP and ANCA-AV-ILD combined with APE. Table 2 showed, in the ILD subgroups, ANCA-AV-ILD, HP, IPF and CTD-ILD respectively had higher VTE incidence rates of 7.9%, 3.6%, 3.5% and 3.0%. Patients with IPF had the same incidence of PTE and DVT (1.6%). Patients with CTD-ILD had a higher incidence of DVT compared to PTE of 2.3% and 0.4%**,** while PTE was more common in patients with HP compared to DVT at 2.4% and 0.8% (Table 2).

**Table 2:** The incidence of VTE in different types of ILD

Characteristics	Total, *n* = 5009 (%)	IPF, *n* = 5210 (%)	NSIP, *n* = 407 (%)	CTD-ILD,* n* = 1576 (%)	COP, *n* = 534 (%)	HP, *n* = 250 (%)	ANCA-AV–ILD, *n* = 63 (%)	Others, *n* = 1669 (%)
VTE	129(2.6)	18(3.5)	10(2.5)	48(3.0)	13(2.4)	9(3.6)	5(7.9)	26(1.6)
Only PTE	34(0.7)	8(1.6)	5(1.2)	7(0.4)	1(0.2)	6(2.4)	2(3.2)	5(0.3)
Only DVT	80(1.6)	8(1.6)	2(0.5)	37(2.3)	9(1.7)	2(0.8)	2(3.2)	20(1.2)
Concomitant PTE and DVT	15(0.3)	2(0.4)	3(0.7)	4(0.3)	3(0.6)	1(0.4)	1(1.6)	1(0.1)

*ILD* interstitial lung disease, *IPF* idiopathic pulmonary fibrosis, *NSIP* nonspecific interstitial pneumonia, *CTD-ILD* connective tissue disease-associated ILD, *COP* cryptogenic organized pneumonia, *HP* hypersensitivity pneumonitis, *ANCA-AV-ILD* Anti-Neutrophil Cytoplasmic Antibodies -associated vasculitis related ILD, *VTE* venous thromboembolism, *PTE* pulmonary thromboembolism, *DVT* deep vein thrombosis

Among 1576 patients with CTD-ILD, the incidence of VTE was 3.0%, PTE was 0.4%, concomitant PTE and DVT was 0.3% and DVT was 2.3%**,** respectively. Table 3 showed the incidence of VTE in patients with CTD-ILD in order of incidence: SLE, 2 (8.7%); IIM, 27 (4.0%); RA, 9 (3.6%); SS, 6 (1.5%); OCTD-ILD, 4 (1.7%), respectively (Table 3).

**Table 3:** The composition of VTE in different types of CTD-ILD

Characteristics	Total, *n* = 1576, (%)	IIM-ILD,* n* = 680 (%)	SS-ILD, *n* = 388 (%)	RA-ILD, *n* = 249 (%)	SLE-ILD, *n* = 23 (%)	OCTD-ILD, *n* = 236 (%)
VTE	48(3.0)	27(4.0)	6(1.5)	9(3.6)	2(8.7)	4(1.7)
Only PTE	7(0.4)	3(0.4)	0(0)	1(0.4)	1(4.3)	2(0.8)
Only DVT	37(2.3)	22(3.2)	6(1.5)	7(2.8)	0(0)	2(0.8)
Concomitant PTE and DVT	4(0.3)	2(0.3)	0(0)	1(0.4)	1(4.3)	0(0)

*VTE* venous thromboembolism, *CTD-ILD* connective tissue disease-associated ILD, *IIM-ILD* idiopathic inflammatory myopathies related ILD, *SS-ILD* Sjogren’s syndrome related ILD, *RA-ILD* rheumatoid arthritis related ILD, *SLE-ILD* systemic lupus erythematosus related ILD, *PTE* pulmonary thromboembolism, *DVT* deep vein thrombosis

### Demographics of ILD patients developed VTE

**Fig. 3:**
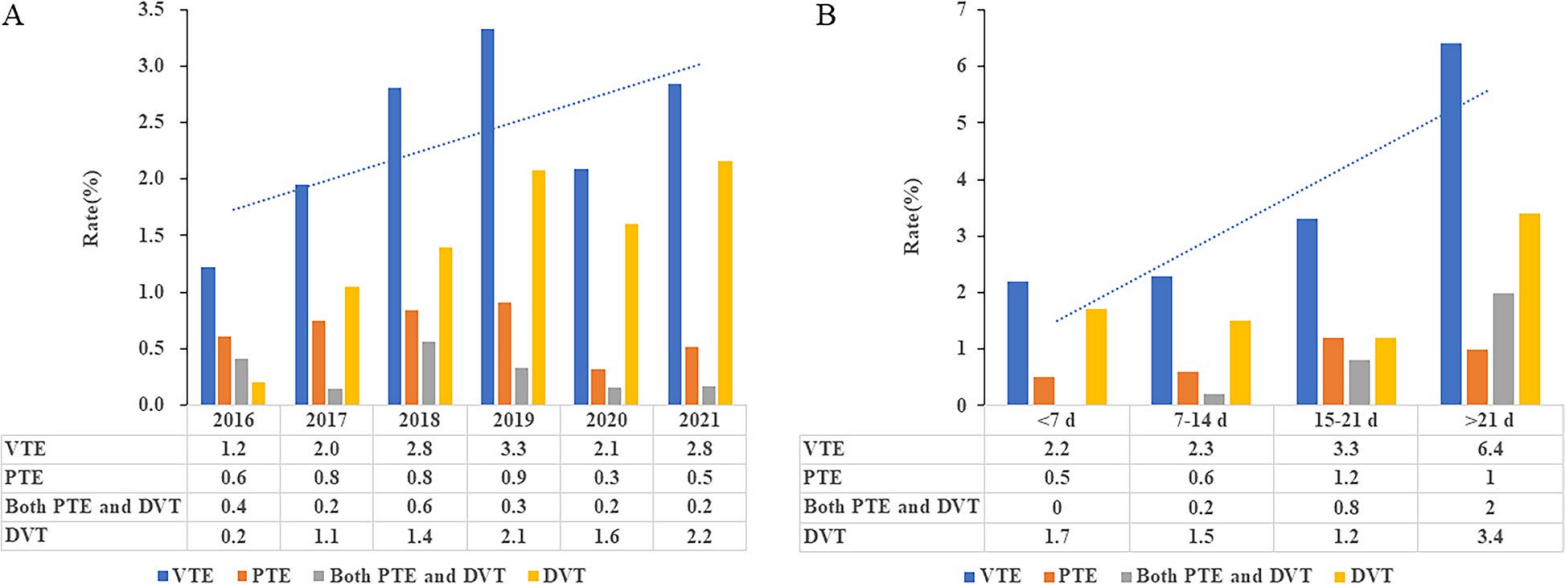
Incidence of VTE in ILD. **A** Trends in annual incidence of VTE in ILD. **B** Changes in the incidence of VTE in ILD with the length of time in hospital. ILD, interstitial lung disease; IPF, idiopathic pulmonary fibrosis; NSIP, nonspecific interstitial pneumonia; CTD-ILD, connective tissue disease-associated ILD; COP, cryptogenic organized pneumonia; HP, hypersensitivity pneumonitis; ANCA-AV-ILD, Anti-Neutrophil Cytoplasmic Antibodies -associated vasculitis related ILD; VTE, venous thromboembolism; PTE, pulmonary embolism; DVT, deep vein thrombosis

Table 3 indicated, among those ILD patients who developed VTE, the incidence of concomitant PTE and DVT, PTE and DVT respectively were 11.6%, 26.4% and 62.0%. The median age of patients with VTE was 66 years (IQR, 60 to 74 years) and VTE occurred more commonly in female with ILD, including a combination of PTE and DVT, PTE and DVT. The three most frequent complications were autoimmune diseases, lung infection and respiratory failure with the incidence of 38.8%, 37.2% and 26.4%, respectively, which followed by diabetes and hypertension (Table 4). The prevalence of VTE in ILD was increasing from 1.2% in 2016 to 2.8% in 2021 (Fig. 3A). In patients with APE, 2.1% of patients were at high-risk group, 14.9% of patients were at intermediate-high risk group and the remaining 83.0% of patients were at intermediate-low risk group. Meanwhile, the incidence of VTE increased significantly as the number of days in hospital increased, from 2.2% in patients with a length of stay of less than 7 days in ILD to 6.4% in patients with a length of stay of more than 21 days (Fig. 3B).

**Table 4:** Demographic characteristics of VTE

Characteristics	VTE, *n* = 129 (%)	Only PTE, *n* = 34 (%)	Only DVT, *n* = 80 (%)	Concomitant PTE and DVT, *n* = 15 (%)
**Population**	**12**9**(100.0)**	**35(27.1)**	**80(62.0)**	**16(12.4)**
**Age, Median (IQR) years**	66(60–74)	69(62–76)	65(58–72)	72(62–81)
< 60	29(22.5)	7(20.6)	21(26.3)	1(6.7)
60–69	50(38.8)	13(38.2)	32(40.0)	5(33.3)
70–79	36(27.9)	9(26.5)	22(27.5)	5(33.3)
≥ 80	14(10.9)	5(14.7)	5(6.3)	4(26.7)
**Male**	59(45.7)	16(47.1)	37(46.3)	6(40.0)
**Predisposing factors**				
**Strong risk factors**				
Myocardial infarction	2(1.6)	1(2.9)	1(1.3)	0(0)
Atrial fibrillation	2(1.6)	1(2.9)	1(1.3)	0(0)
Trauma/surgery	2(1.6)	0(0)	2(2.5)	0(0)
**Moderate risk factors**				
Autoimmune diseases	50(38.8)	8(23.5)	37(46.3)	5(33.3)
Respiratory failure	34(26.4)	11(32.4)	17(21.3)	6(40.0)
Lung infection	48(37.2)	16(47.1)	26(32.5)	6(37.5)
Urinary tract infections	0(0)	0(0)	0(0)	0(0)
Inflammatory bowel disease	0(0)	0(0)	0(0)	0(0)
Malignancies	8((6.2)	1(2.9)	6(7.5)	1(6.7)
**Weak risk factors**				
Diabetes	26(20.2)	5(14.37)	20(25.0)	1(6.7)
Hypertension	26(20.2)	7(20.6)	16(20.0)	3(20.0)
Varicose veins	3(2.3)	0(0)	3(3.8)	0(0)

*ILD* interstitial lung disease, *VTE* venous thromboembolism, *PTE* pulmonary thromboembolism, *DVT* deep vein thrombosis, *IQR* interquartile range

### Identification of risk factors for VTE occurrence in ILD

**Fig. 4:**
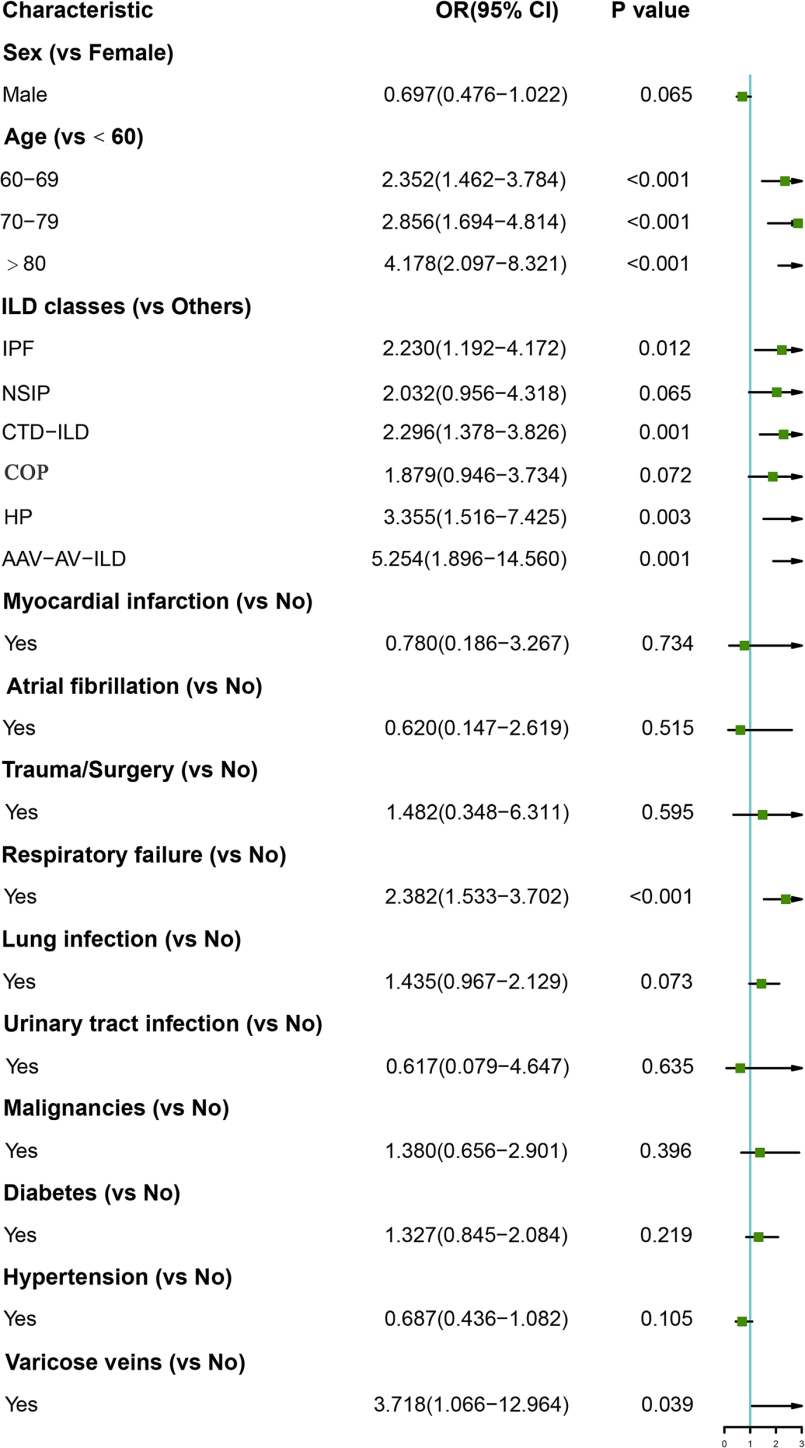
Logistics regression analysis of independent risk factors for the occurrence of VTE. ILD, interstitial lung disease; IPF, idiopathic pulmonary fibrosis; NSIP, nonspecific interstitial pneumonia; CTD-ILD, connective tissue disease-associated ILD; COP, cryptogenic organized pneumonia; HP, hypersensitivity pneumonitis; ANCA-AV-ILD, Anti-Neutrophil Cytoplasmic Antibodies -associated vasculitis related ILD; VTE, venous thromboembolism; PTE, pulmonary thromboembolism; DVT, deep vein thrombosis; OR, odds ratio

Logistic regression analysis further revealed that age, ILD subtypes, respiratory failure and varicose veins were associated with VTE in patients with ILD. Age ≥ 80 years (OR 4.178, 95% CI 2.097–8.321, *P* < 0.001), ILD subtypes [ such as IPF (OR 2.230, 95% CI 1.192–4.172, *P* = 0.012), CTD-ILD (OR 2.296, 95% CI 1.378–3.826, *P* = 0.001), HP (OR 3.355, 95% CI 1.516–7.425, *P* = 0.003), AAV-AV-ILD (OR 5.254, 95% CI 1.896–14.560, *P* = 0.001)], respiratory failure (OR 2.382, 95% CI 1.533–3.702, *P* < 0.001) and varicose veins (OR 3.718, 95% CI 1.066–12.964, *P* = 0.039) were independent risk factors for the occurrence of VTE (Fig. 4).

## Discussion

In our single-center research, the incidence of VTE in patients with ILD was 2.6%, including 1.6% DVT, 0.7% PTE, both PTE and DVT 0.3%. The incidence of VTE in patients with ANCA-AV-ILD, HP, IPF and CTD-ILD respectively was 7.9%, 3.6%, 3.5% and 3.0%. 83.0% of patients with ILD combined with PTE were predominantly at low to moderate risk. Furthermore, the advanced age, different ILD types, respiratory failure and varicose veins were independent risk factors for the development of VTE.

This study firstly detailed analysis of the incidence of VTE in patients with ILD. Besides the traditional risk factors for VTE such as surgery, cancer and the use of certain drugs, chronic inflammation has recently been considered as risk factor for VTE. Increased incidence of VTE has been observed in a variety of chronic inflammatory diseases such as rheumatoid arthritis, systemic vasculitis and inflammatory myositis [20–22]. ILD is a diffuse lung disease involving the alveoli and interstitial lung and patients may have a higher risk of VTE because of the increased systemic inflammatory burden [8, 23–25]. Several previous studies have reported the association of ILD with the occurrence and recurrence of VTE [26–28]. Most of the studies concentrated on IPF, and a meta-analysis revealed a 2.11-fold risk of VTE in patients with IPF [15]. Apart from potential risks such as inflammation, this higher incidence of VTE in IPF may be associated with hormone exposure [29]. Also, as a subtype of ILD with a poor prognosis, IPF frequently suffers from respiratory failure and bed rest in the late stages [30], all of which may contribute to the high VTE rate. In our study, patients with IPF had a higher median age of 67 years (IQR, 61 to72 years) and were more susceptible to combined respiratory failure (17.3%) and malignancy (5.1%) compared to other types of ILD. Meanwhile, higher incidence of VTE in IPF was also observed of 3.5%. A retrospective study by Sobiecka M et al. [31] on 441 patients with ILD showed that HP and IPF had similar VTE incidence rates of 3.3% and 4.6%, respectively. We further demonstrated similar VTE incidence between these two ILD types (HP, 3.6%; IPF, 3.5%). The relatively low incidence of VTE in IPF we reported compared to that study is considered to to be due to the fact that we focused on the incidence of VTE during hospitalization. As the length of the length of time in hospital increases, the incidence of VTE in patients with ILD increases from 2.2% at less than 7 days’ hospital stay to 6.4% at more than 21 days’ hospital stay. Some interventions such as glucocorticoid exposure are also risk factors for VTE [32, 33], and the limited follow-up time underestimates the effect of pharmacological interventions on VTE occurrence. Our study found the same incidence of PTE and DVT in IPF of 1.6%, while in CTD-ILD, the incidence of DVT was higher than PTE of 2.3% and 0.4%, respectively [16].

Further analysis revealed that VTE in ILD subtypes were independent risk factors for the development of VTE. The risk of VTE in patients with ILD was significantly increased by advanced age, respiratory failure and varicose veins, which were also identified as risk factors for VTE in the 2019 European Respiratory Society guidelines [30]. The fact that ILD tends to occur in older patients and that ILD with a progressive phenotype such as IPF tends to have respiratory failure at the end of the disease are factors that contribute to the high incidence of VTE in the patients of ILD.

Our study still has some limitations. First, this was a single-center, retrospective, cross-sectional study and there may be selection bias. Furthermore, some of the patients lost to follow-up may have developed VTE, which may lead to an underestimation of the incidence of VTE in patients with ILD. Therefore, large multicenter prospective studies and the long-term follow-up are particularly essential for assessment of VTE incidence in patients with ILD.

## Conclusion

Among 5009 Chinese patients with different ILD subtypes, the incidence of VTE, DVT and PTE respectively was 2.6%, 1.6% and 0.7%. ANCA-AV-ILD had the highest incidence rate of 7.9%. HP and IPF had similar incidence rates of 3.6% and 3.5%, respectively, followed by CTD-ILD. Advanced age, ILD subtypes, respiratory failure and varicose veins were independent risk factors for the occurrence of VTE. Further research is needed to understand the role of systematic screening for VTE in patients with ILD.

## Data Availability

The original contributions presented in the study are included in the article/Supplementary Materials; further inquiries can be directed to the corresponding author.

## Abbreviations

ILD: Interstitial lung disease
IPF: Idiopathic pulmonary fibrosis
NSIP: Nonspecific interstitial pneumonia
CTD-ILD: Connective tissue disease-associated ILD
COP: Cryptogenic organized pneumonia
HP: Hypersensitivity pneumonitis
ANCA-AV: Anti-Neutrophil Cytoplasmic Antibodies -associated vasculitis
IIM: Idiopathic inflammatory myopathies
SS: Sjogren’s syndrome
RA: Rheumatoid arthritis
SLE: Systemic lupus erythematosus
VTE: Venous thromboembolism
PTE: Pulmonary thromboembolism
DVT: Deep vein thrombosis. CTPA: CT pulmonary arteriography
HRCT: High resolution computed tomography
